# Expression of Enzymatically Inactive Wasp Venom Phospholipase A1 in *Pichia pastoris*

**DOI:** 10.1371/journal.pone.0021267

**Published:** 2011-06-23

**Authors:** Irina Borodina, Bettina M. Jensen, Tim Wagner, Maher A. Hachem, Ib Søndergaard, Lars K. Poulsen

**Affiliations:** 1 Center for Microbial Biotechnology, Institute of Systems Biology, Technical University of Denmark, Kgs. Lyngby, Denmark; 2 Allergy Clinic, Dermato-Allergological Dept. K, CUH-Gentofte, Rigshospitalet Dept 7551, København Ø, Denmark; 3 Enzyme and Protein Center, Institute of Systems Biology, Technical University of Denmark, Kgs. Lyngby, Denmark; Cinvestav, Mexico

## Abstract

Wasp venom allergy is the most common insect venom allergy in Europe. It is manifested by large local reaction or anaphylactic shock occurring after a wasp sting. The allergy can be treated by specific immunotherapy with whole venom extracts. Wasp venom is difficult and costly to obtain and is a subject to composition variation, therefore it can be advantageous to substitute it with a cocktail of recombinant allergens. One of the major venom allergens is phospholipase A1, which so far has been expressed in *Escherichia coli* and in insect cells. Our aim was to produce the protein in secreted form in yeast *Pichia pastoris*, which can give high yields of correctly folded protein on defined minimal medium and secretes relatively few native proteins simplifying purification.

Residual amounts of enzymatically active phospholipase A1 could be expressed, but the venom protein had a deleterious effect on growth of the yeast cells. To overcome the problem we introduced three different point mutations at the critical points of the active site, where serine137, aspartate165 or histidine229 were replaced by alanine (S137A, D165A and H229A). All the three mutated forms could be expressed in *P. pastoris*. The H229A mutant did not have any detectable phospholipase A1 activity and was secreted at the level of several mg/L in shake flask culture. The protein was purified by nickel-affinity chromatography and its identity was confirmed by MALDI-TOF mass spectrometry. The protein could bind IgE antibodies from wasp venom allergic patients and could inhibit the binding of wasp venom to IgE antibodies specific for phospholipase A1 as shown by Enzyme Allergo-Sorbent Test (EAST). Moreover, the recombinant protein was allergenic in a biological assay as demonstrated by its capability to induce histamine release of wasp venom-sensitive basophils.

The recombinant phospholipase A1 presents a good candidate for wasp venom immunotherapy.

## Introduction

Allergy to wasp *Vespula vulgaris* and *Vespula germanica* venom is the most common insect sting allergy in temporate Europe and is a cause of significant morbidity, impairment of life quality and can sometimes be fatal [1]. Epidemiologic studies report prevalence of systemic anaphylactic reactions in general population at 1–1.5% [2], [3]. Patients can be treated by specific immunotherapy (SIT) with venom extract, which is obtained in a tedious and expensive procedure where venom sacs are manually removed from collected wasps and then the extract is partially purified. The extract is subject to composition variation, which can cause adverse effects during treatment; furthermore it contains a number of non-allergenic proteins [4]. Using recombinant allergens as a vaccine instead of the venom extract could improve the treatment of wasp venom allergies by providing a cheaper, well-characterized, and composition-consistent vaccine. Additionally the vaccine components could be combined differently to match individual patients' sensitization profiles.

One *V. vulgaris* sting injects around 1.7–3.1 µg of venom, in which the most abundant allergenic proteins (major allergens) are phospholipase A1 (Ves v 1.0101), hyaluronidase (Ves v 2.0101) and antigen 5 (Ves v 5.0101), accounting for correspondingly 3.3%, 1.5% and 8.1% of the total venom protein [5]. A close homologue to hyaluronidase, though without enzymatic activity, allergen Ves v 2.0201 has been found [6], [7]. Recently IgE reactivity and basophils activation has also been shown for a high-molecular mass venom component, 100 kDa dipeptidyl peptidase IV (Ves v 3.0101) [8]. Allergens from *V. vulgaris* have been recombinantly expressed in various hosts as *E. coli*, insect cells and yeast species.

Antigen 5, a 23 kDa non-glycosylated protein with so far unknown function, has been expressed in *E. coli* [9], [10], *P. pastoris* [9], insect cells [11] and recently also on the surface of yeast *Saccharomyces cerevisiae* [12]. Antigen 5 produced in *P. pastoris* has recently become commercially available for diagnostic purposes in ImmunoCAP format (Phadia, Sweden).

Hyaluronidase, 45-kDa glycosylated protein, catalyzing hyaluronic acid degradation and thus facilitating spreading of venom components in the tissue after sting, has been expressed in *E. coli* [13], [14] and in insect cells [15]. The protein expressed in *E. coli* did not obtain enzymatic activity after refolding procedure [14] and had a lower reactivity towards antibodies specific for the native hyaluronidase, indicating that parts of the discontinuous epitopes were lost due to improper folding [13]. It has been hypothesized that glycosylation is important for enzymatic activity and possibly also for correct folding of hyaluronidase [16]. The importance of hyaluronidase for allergic response to wasp venom is probably low as Ves v 2 - specific antibodies are mainly directed towards cross-reactive carbohydrate determinates [15], [17], which are believed to be of low (if any) clinical significance [18].

Phospholipase A1, a 33.4 kDa non-glycosylated protein, removes the 1^st^ acyl group from phospholipids and thus causes damage to cell membranes. Phospholipase A1, expressed in *E. coli* had a lower binding to antibodies specific for the native phospholipase A1 than the native phospholipase A1, suggesting that the recombinant phospholipase A1 was not correctly folded [13]. Enzymatically active and an inactivated variant with two mutations in the putative active site (S137G and D165A) have been expressed in insect cells, both variants were biologically active [11].

While insect cells can provide allergens useful for diagnostic tests [11], [19], the system is less suited for making proteins for therapeutic applications because of low yields, difficulties with scale-up, complex purification process and legal issues. In spite of the long history of baculovirus expression system, only one baculovirus-derived product has been approved by Federal Drug Administration (FDA) so far, namely Cervarix, manufactured by GlaxoSmithKline (UK) [20]. An alternative expression system for inexpensive protein secretion is yeast, where particularly *P. pastoris* has been extensively used recently with several products in the clinical trials pipeline [21], [22] and one FDA-approved product – Kalbitor (Dyax, USA) [20].

The aim of this study was to express enzymatically inactivated variants of phospholipase A1 from *V. vulgaris* in methylotrophic yeast *P. pastoris*, which is well-suited for industrial-scale fermentations due to strain stability, high level of foreign protein secretion, ability to grow to high cell densities on defined minimal medium and low level of secretion of native proteins.

## Materials and Methods

### Ethics statement

The Ethical Committee for the Capital Region of Denmark approved the use of historical blood samples in the project, since the patients had already given their informed consent to the storage and scientific use of their serum samples when their blood was originally drawn. The informed consent procedure was written and verbal. At their first visit to the clinic, the patient is informed that if he or she consents the sample will be stored for possible future analyses relating to his/her treatment and for possible research and development. In case of acceptance by the patient, an informed consent form is signed by the patient, in which it is stated that the patient accept that his/her sample is stored for up to 10 years for these purposes only, and that he/she at any time can have the sample removed from the serum bank.

### Patients

Twenty two patients were chosen based on their serum IgE reactivity with venom extract (i3) in ImmunoCap assay (Phadia), all CAP class 4 or above, and non-detected cross-reactivity with honey bee venom (i1, <0.35 kUa/L). The negative control sera consisted of a pool of 200 non-allergic sera (negative for common inhalation allergens (birch, grass, mugwort, horse, dog, cat, house dust mites and molds) and food allergens (milk, egg white, cod, peanut, soy bean, wheat flour).

### Chemicals

The chemicals were purchased from Sigma-Aldrich and BD Biosciences. Zeocin™ was purchased from Invitrogen. The Anti-His horse radish peroxidase conjugated antibody was a mouse monoclonal IgG1 antibody (Qiagen). The restriction enzymes and T4 DNA ligase were purchased from New England Biolabs Inc. Vespula venom extract (1,000 SQU/µl) used in ELISA was a kind gift of Jørgen Nedergaard Larsen (ALK Abelló, Hørsholm, Denmark). Vespula venom extract used in histamine release assay was from ALK Abelló and contained 136 µg/ml protein. The primers were ordered from Eurofins MWG Operon (Germany).

### Strains and plasmids

The *E. coli* strain used for cloning was DH5α (F^−^Φ80*lac*ZΔM15 Δ(*lac*ZYA-*arg*F)U169 *deo*R *rec*A1 *end*A1 *hsd*R17(r_k_^−^, m_k_^+^) *pho*A*sup*E44 *thi*-1 *gyr*A96 *rel*A1 λ-). The *P. pastoris* strains used for protein expression were X33 (Mut^+^) and KM71H (Mut^S^, *arg4 aox1::ARG4*) (Invitrogen). Vector pPICZalphaA was also purchased from Invitrogen. For longer storage the *E. coli* strains were stored in LB medium with 25% glycerol and yeast strains were stored in YPD (1% yeast extract, 2% peptone, 2% dextrose) with 15% glycerol at −80°C.

### Cloning and mutation of phospholipase A1 gene

The *Vesv1* gene encoding phospholipase A1 was previously cloned from local Danish *V. vulgaris* insects [12]. The gene was codon-optimized using online tool from Mr.Gene GmbH (Germany) and synthesized by the same company. The gene was amplified with vesv1_fw and vesv1_rv primers, the fragment was purified from the 1% agarose gel, digested with *Xho*I and *Xba*I and ligated into pPICZαA plasmid, digested with the same enzymes and gel-purified. The ligation mixture was transformed into *E. coli* DH5α cells and the transformants were selected on low salt Luria-Bertani (LB) medium with 25 µg/ml zeocin. The presence of the insert in the plasmid was tested by colony PCR and the correct transformants were grown overnight in liquid low-salt LB medium with zeocin selection after which the plasmids were isolated. The correct cloning of the *Vesv1* gene was confirmed by restriction analysis and sequencing (StarSEQ, Germany).

The plasmid was mutated by site-directed mutagenesis using QuickChange® II XL Site-Directed Mutagenesis kit from Stratagene (USA). The primers used in pairs to generate three mutations are given in Table 1.

**Table 1:** List of primers.

Primer Name	Sequence	Application
vesv1_fw	*Xho*I5′-CATCCTCGAGAAAAGA GGACCAAAATGCCCATTC	Amplification of *vesv1* gene for cloning into expression vector pPICZαA
vesv1_rv	5′-GCACGTCTAGAGC GATGATTTTTCCTTTGTTGTTAC	
vesv5_fw	5′- CATCCTCGAGAAAAGA AACAATTATTGTAAAATAAAATG	Amplification of *vesv5* gene for cloning into expression vector pPICZαA
vesv5_rv	5′- TACTTCTAGAGC CTTTGTTTGATAAAGTTC	
Vesv1_S137A_fw	5′-CAGATTGATCGGACAC**GCT**TTGGGTGCTCACGC	Change of serine137 to alanine
Vesv1_S137A_rv	5′-GCGTGAGCACCCAA**AGC**GTGTCCGATCAATCTG	
Vesv1_D165A_fw	5′-GAGATCATCGGATTG**GCC**CCTGCTAGACCTTCT	Change of aspartate165 to alanine
Vesv1_D165A_r	5′-AGAAGGTCTAGCAGG**GGC**CAATCCGATGATCTC	
Vesv1_H229A_fw	5′-CTTCTCCGAAGTTTGCTCT**GCT**AGTAGAGCCGTCATTTAC	Change of histidine229 to alanine
Vesv1_H229A_rv	5′-GTAAATGACGGCTCTACT**AGC**AGAGCAAACTTCGGAGAAG	
AOX3	5′-GCAAATGGCATTCTGACATC	Colony PCR to test the integration of inserts into the pPICZαA vector and RT-PCR
AOX5	5′-GACTGGTTCCAATTGACAAG	

*The altered triplets are emphasized in the primers used for directed point mutagenesis of vesv1 gene. Recognition sites for restriction endonucleases XbaI (TCTAGA) and XhoI (CTCGAG) are underlined.*

### Yeast transformation

The 4 constructed plasmids were linearized with *Sac*I and transformed into *P. pastoris* strains X33 and KM71H using the optimized electroporation protocol by Wu and Letchworth [23]. The transformants were selected on YPDS medium (same as YPD, but with additionally 1 M sorbitol) with 100 µg/ml zeocin and streak-purified. The integration of the plasmids in the yeast genome was confirmed by colony PCR.

### Cultivation and induction of yeast cells

For X33 Mut^+^ strains a single colony was inoculated into 50 ml BMGY (buffered minimal glycerol medium, recipe from Invitrogen) in 500-ml baffled shake flask and grown for 16 hours at 30°C with shaking at 150 rpm. The OD_600_ of the culture was measured and a suitable volume was centrifuged to give on resuspension in BMMY (buffered minimal methanol medium) an OD_600_ of 1. The BMMY medium contained 1% methanol and additionally 1% casamino acids. 25 ml of cells resuspended in BMMY was transferred to a 500-ml baffled shake flask and induction was carried out either at 20°C or 30°C at 150 rpm rotation for 72 hours with daily addition of 1% methanol.

For Mut^S^ strains a single colony was inoculated into 200 ml BMGY medium and grown as for Mut+ strains for 24 hours. The culture was centrifuged and resuspended in 50 ml BMMY medium containing 0.5% methanol, 0.5% glycerol and 1% casamino acids. The cell suspension was divided into 2 shake flasks, 25 ml in each, which were induced at 20°C and 30°C for 72 hours with daily addition of 1% of 1∶1 mixture (v/v) of glycerol and methanol.

During the cultivation 1 ml samples were withdrawn into chilled 1.5 ml eppendof tubes and centrifuged at 16,000×g for 1 min. The supernatant and cell pellet were stored at −20°C until analysis.

To generate fermentation broth for purification larger volumes were used as following. Single colonies of Mut^S^ strains expressing mutated version of phospholipase A1 were inoculated in 50 ml BMGY medium in 500-ml volume shake flasks with baffles and grown at 30°C for 16 hours with 150 rpm shaking. The whole culture was used to inoculate 500 ml BMGY in a 2-L flask with baffles and grown at the same conditions as before for 24 hours. The cells were recovered by centrifugation, resuspended in 100 ml BMMY medium with 0.5% methanol and 0.5% glycerol and transferred to a 500-ml shake flask with baffles. The induction was performed for 96 hours at 20°C with 150 rpm agitation and daily addition of 1% 1∶1 methanol∶glycerol mixture. At the end of the fermentation the biomass reached around ∼130 g wet weight biomass/L. The flasks were cooled on ice, the broth was centrifuged at 8,000×g for 10 min at 4°C and decanted. 100 µl of protease inhibitor cocktail (Sigma) was added and the broth was stored at −20°C until purification.

### Cloning and expression of antigen 5

The gene encoding antigen 5, *Vesv5*, was cloned from was venom sac RNA into a TOPO vector (Invitrogen) [12]. The gene part encoding the mature peptide was amplified using primers vesv5_fw and vesv5_rv (Table 1) and cloned into pPICZαA plasmid as described above for *Vesv1* gene. The resulting plasmid pPICZαA_vesv5 was transformed into *P. pastoris* KM71H strain, cultivated and the protein purified in the same manner as described above for Ves v 1.

### RNA isolation and RT-PCR

The yeast strains were cultivated as described above. 24 hours after the start of induction the OD_600_ of the cultures was measured and the samples corresponding to 5·10^7^ cells (assuming OD_600_ of 1 correspond to 10^7^ cells per ml) were quickly withdrawn into chilled 1.5 ml eppendorf tubes and briefly centrifuged at 16,000×g for 20 s. The supernatant was removed and the cell pellet resuspended in 200 µl RNALater solution (Ambion). The cells were incubated on ice for 30 min, then briefly centrifuged, the RNALater solution removed and the cells snap-frozen in liquid nitrogen and stored at −80°C until RNA isolation. The total RNA was isolated using RNAeasy Mini kit from Qiagen, the residual DNA was removed using TURBO DNA-free™ DNase from Ambion. The RT-PCR was carried out using Titan One Tube RT-PCR kit from Roche with AOX5 and AOX3 primers (Table 1). To ensure that the product results from RNA and not DNA, reactions with addition of only Taq polymerase instead of reverse transcriptase and DNA polymerase mix were carried out in parallel for all the reactions. The products of the reactions were analyzed on 1% agarose gel strained with SYBR-SAFE (Invitrogen).

### SDS-Polyacrylamide Gel Electrophoresis

The gel was a pre-cast 4–12% NuPAGE® Novex® Bis-Tris electrophoresis gel from Invitrogen. The protein size marker was unstained or pre-stained PageRuler™ protein ladder from Fermentas (Germany). The samples were mixed with NuPAGE® LDS sample buffer (4×) and NuPAGE® reducing agent (10×) and heated at 70°C for 10 min before loading on the gel. The electrophoresis was performed at a constant voltage of 200 V for 35 min. The gel was used for western blot or stained with silver stain (Fermentas, Germany).

### Western blot

For western blot the proteins were elecrophoretically transferred onto 0.2 µm PVDF membrane Amersham Hybond™-P (GE Healthcare) using Mini Trans-Blot® electrophoretic transfer cell (Bio-Rad Laboratories) at 100 V for 1 hour. The transfer was done in 25 mM Tris, 192 mM glycine, and 20% v/v ethanol buffer. The membrane was blocked with the blocking reagent supplied together with Anti-His antibody (Qiagen) overnight, incubated with Anti-His antibody in blocking buffer (1∶1,000) for 1.5 hour, washed with TBST buffer for 10 min three times and with TBS buffer for 5 min once. The bound antibody was detected by chemoluminiscence with Amersham ECL™ Advance Western Blotting Detection Kit (GE Healthcare) and the signal was recorded with camera [Hamamatsu Photonics, Japan].

### Enzymatic assays

Phospholipase A1 enzymatic assay was performed using EnzChek® phospholipase A1 assay kit from Invitrogen according to the manufacturer's protocol with Lecitase® Ultra as a standard. The fermentation broth from the cells transformed with empty plasmids was used as background control. For the purified proteins the storage buffer (PBS with 30% glycerol) was used as background control. All measurements were performed at least in duplicates.

### Purification with Ni-affinity chromatography

The proteins were recovered from the fermentation broth as following. Glycerol was added to the broth to the final concentration of 10% and 3 M NaCl to the final concentration of 300 mM. Detergent Tween 20 was added to the final concentration of 0.05%. The pH of the broth was adjusted to 7.5, the broth was centrifuged at 8,000×g for 15 min at 4°C and filtered through 0.45 µm filter. A 1-ml column was packed with Ni-NTA Superflow resin from Qiagen, washed with MilliQ water for 3 column volumes (CV) and equilibrated with 20 CV of buffer A (50 mM sodium phosphate, 300 mM NaCl, 10 mM imidazole, 10% glycerol, 0.05% Tween 20, pH 7.5) at 1 ml/min flow rate. The broth was loaded on the column at 1 ml/min, the column was washed with at least 20 CV of buffer A and the protein was eluted with buffer B, which differed from buffer A by a higher imidazole concentration of 250 mM. The fractions containing the protein were pooled and desalted on Zeba™ Desalt Spin Columns (Thermo Scientific). Protein concentration was measured by amino acids analysis [24], [25]. The proteins were resuspended in PBS buffer with 30% glycerol and stored at −20°C.

### Quantification of 6-HIS-tagged proteins by ELISA

Maxisorb microtiter plates (Nunc, Denmark) were coated with 100 µl samples diluted 1∶10 or 1∶100 in PBS buffer. The standard curve was made with serial double-fold dilutions of purified rVes v 1 H229A, which concentration was determined by amino acids analysis. The coating was performed at 4°C overnight. The plates were washed with phosphate-buffered saline with 0.1% Tween20 (PBST) and blocked with 200 µl PBSTM (5% skim milk in PBST) for 1 hour at room temperature. The plates were washed with PBST and incubated for 2 hours at room temperature with anti-penta-his HRP-conjugated antibody (Qiagen) diluted 1∶500 in PBSTM. The plates were washed with PBST after which 100 µl of TMB ONE liquid substrate (Kem-En-Tek Diagnostics A/S, Denmark) reagent was added to each well and the reaction was allowed to proceed for 10 min at room temperature in the dark. The reaction was terminated by addition of equal volume of 0.3 M H_2_SO_4_. And the absorbance was measured at 450 nm using Synergy reader (BioTek).

### Quantification of IgE binding and inhibition by Enzyme Allergo-Sorbent Test (EAST)

Maxisorb microtiter plates were coated with purified recombinant allergens at the concentration 3 µg/ml in DPBS buffer (Gibco) or with venom extract diluted with DPBS to the final concentration of 10,000 SQ/ml. The coating was performed at room temperature overnight. The plates were washed with ELISA buffer (Pharmacy of the Danish Capital Region), blocked with blocking/sample buffer (1% BSA, 0.1% Tween 20 in DPBS) for 2 hours at 37°C, washed again with ELISA buffer and incubated with shaking at room temperature overnight with patients serum diluted 1∶10 in sample buffer. The plates were washed, incubated with 1∶250 Biotin Mouse a-Human IgE (BD) in sample buffer for 2 hours at 37°C, washed, incubated with 1∶2000 ExtrAvidin-Peroxidase (Sigma) in sample buffer for 30 min at room temperature, washed again and developed with OPD reagent (Dako) as recommended by manufacturer.

For inhibition assays the serum was pre-incubated with recombinant allergen or wasp venom in sample buffer (1∶20) at 4°C overnight before loading on the coated plates.

### Histamine release assay

Peripheral blood mononuclear cells (PBMCs) from buffy coat blood (non-allergic donor, anti-IgE responsive cells) were isolated by Lymphoprep gradient centrifugation. IgE was stripped off the basophils by a rebound in pH from 7.4 to 3.55 and back to 7.4. The PBMCs were then incubated 1 hour with wasp patient or control pool (non-allergic) serum to re-sensitize the basophils, which subsequently were mixed with erythrocytes and challenged in glass fiber coated microtiter plates (RefLab, Copenhagen, Denmark) with wasp venom extract or with purified recombinant allergens. Released histamine bound to the glass fibers was coupled to o-phtahaldialdehyde, stabilized by HCLO_4_, and measured fluorometrically as described previously [26]. Results were expressed as percentage of total cellular histamine content and were considered positive when >10%.

### Protein analysis by MALDI-TOF

Protein spots were picked from Coomassie-stained gels. Tryptic digestion was carried out as previously described [27]. The peptide solution was applied on Anchorchip targetTM (Bruker Daltonics) using CHCA as matrix. MS analysis was performed on a MALDI-TOF-TOF Ultraflex II in positive ion reflector mode and spectra were processed and analyzed using the software FlexAnalysis and BioTools (Bruker Daltonics). Database searching was carried out for each individual sample using an in-house MASCOT server (Matrix Science, London, UK) to search NCBInr (ftp://ftp.ncbi.nih.gov/blast/db/). The search criteria were selected as following: peptide tolerance ±50 ppm, fixed modifications – carbamidomethyl (C), variable modifications – oxidation (M), allow up to 1 missed tryptin cleavages.

## Results

### Cloning and mutation of phospholipase A1

**Figure 1:**
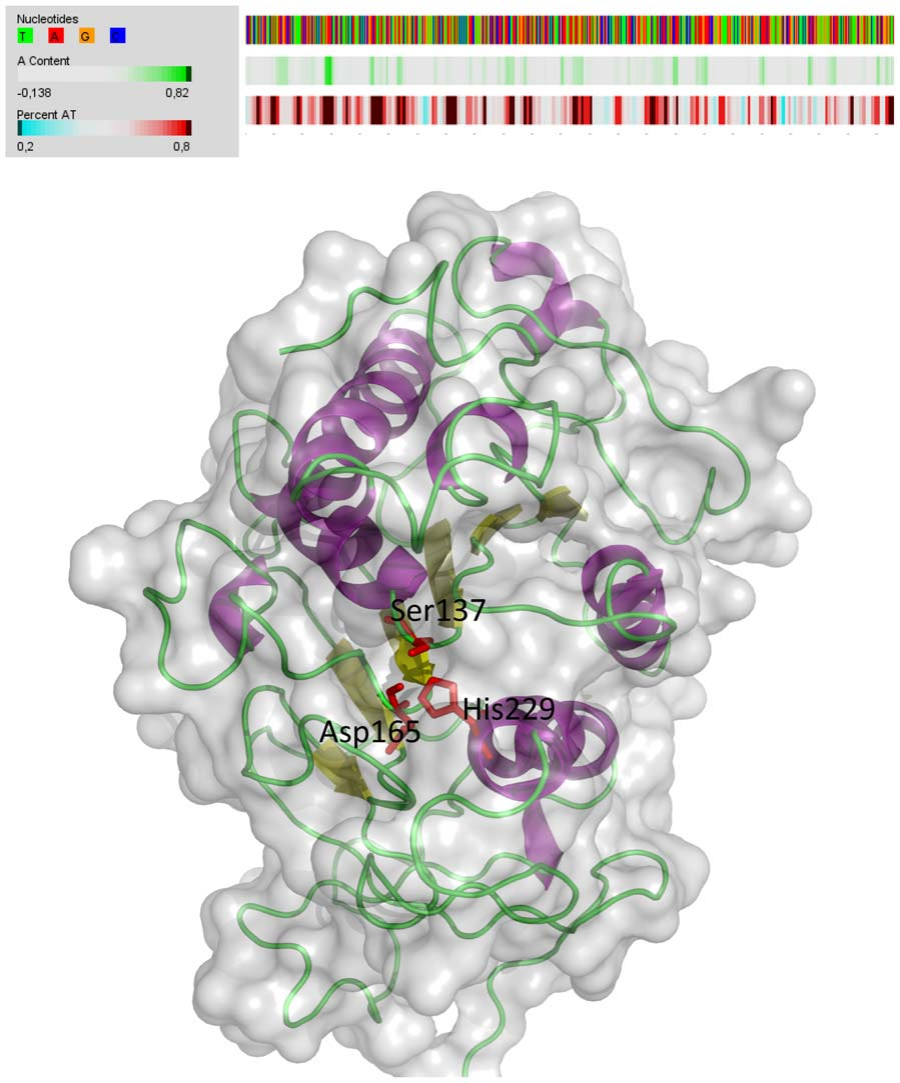
AT-content of *Vesv1* gene and predicted tertiary structure of Ves v 1 protein. Plotting of the AT-content of the Vesv1 gene was performed using Gene Atlas utility (www.cbs.dtu.dk). The dark brown areas on the third line show DNA stretches with AT content above 80%. The 3D structure of the Ves v 1 protein was generated using Geno3D (http://geno3d-pbil.ibcp.fr) using rat pancreatic lipase related protein 2 (1BU8) as a template (29.4% identity, 43.7% similarity) [32]. The structure was visualized in PyMOL 1.3 (http://www.pymol.org). The mutated amino acids are shown in red.

We previously isolated a *Vesv1* gene encoding wasp venom phospholipase A1 from a local Danish species of *V. vulgaris*. On the inspection of the sequence we found that it contained some longer AT-rich stretches, which could serve as premature termination signals to yeast (Figure 1). To avoid incomplete transcription of the gene in the recombinant host we optimized the gene sequence using online tool provided by Mr.Gene GmbH (Germany). During the optimisation the most common *P. pastoris* codons were preferentially used, though synonymous codons were used as well to avoid excessive repetitions and restriction sites. We avoided restriction sites necessary for cloning and plasmid linearization as well as yeast splice donor sites, poly(A)-sites, TATA-boxes, RBS, and −35 prokaria boxes.

The synthetic gene was cloned in expression plasmid pPICZαA after the strong methanol-inducible alcohol oxidase 1 (AOX1) promoter and in-frame with the α-mating factor pre-propeptide for secretion from the cells. The genes were fused with c-myc and 6×HIS tags at the C-terminus for easy detection and purification. The resulting plasmid was mutated to substitute three different amino acids from the predicted active site, serine137, aspartate165 and histidine229 to alanine (Figure 1). The 3D modelling of the mutated Ves v 1 variants was performed as well and the mutated versions aligned well with the wild-type model, indicating that the introduced mutations should not cause significant changes in the 3D structure of the molecule. The resulting plasmids with wild-type and mutated variants of the *Vesv1* gene were introduced into two *P. pastoris* strains: wild type X33 strain with an active AOX1 gene giving fast growth on methanol (Mut^+^ phenotype) and strain KM71H with a deletion in AOX1 gene resulting in slow growth on methanol (Mut^S^ phenotype). Induction was performed in complex medium in shake flasks at 20° or 30°C. The induction of KM71H strain was performed with a 1∶1 mixture of glycerol and methanol because we found in preliminary experiments that it gave better final product titer than induction with pure methanol (data not shown).

### Expression of active phospholipase A1 and its mutated variants

**Figure 2:**
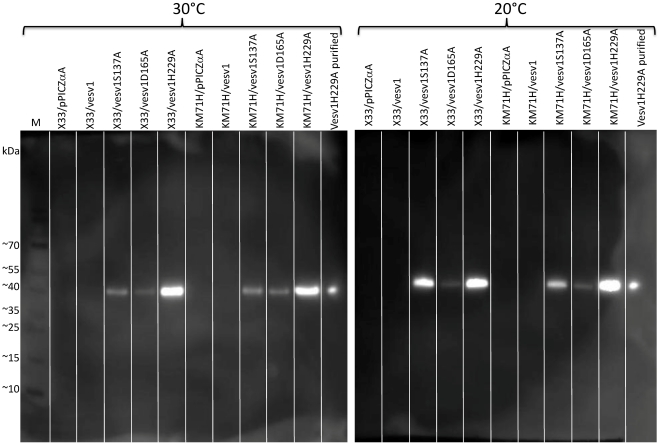
Analysis of fermentation samples on Western blot. 10 µl of fermentation broth after 3-day induction on methanol at 20 and 30°C were analyzed by Western blot with anti-penta-His antibody. For several strains up to 4 transformants were tested and no significant difference between clones was observed. The marker is a pre-stained PageRuler (Fermentas, Germany). Lot-to-lot variation of the apparent molecular weight of pre-stained proteins in the ladder is ∼5%.

**Figure 3:**
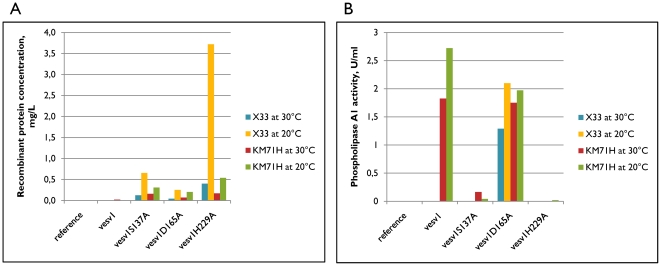
Recombinant proteins concentration and enzymatic activity. Concentration of recombinant protein in fermentation broth (same samples as on Figure 2) were measured by ELISA with anti-penta-His antibody (A). Phospholipase A1 enzymatic activity was measured in a fluorometric commercial assay (Invitrogen) (B).

Fermentation broth was analyzed for the presence and size of recombinant proteins using Western blot (Figure 2). The concentrations of recombinant proteins in the broth were measured by ELISA and phospholipase A1 activity was analyzed in enzymatic assay (Figure 3).

While phospholipase A1 enzymatic activity was detected in the broth of cultures expressing a wild-type version of *Vesv1* gene, the protein could not be detected on Western blot under any expression conditions. The reason could be too low expression levels of the protein or partial degradation of the protein with a loss of the C-terminal tag.

**Figure 4:**
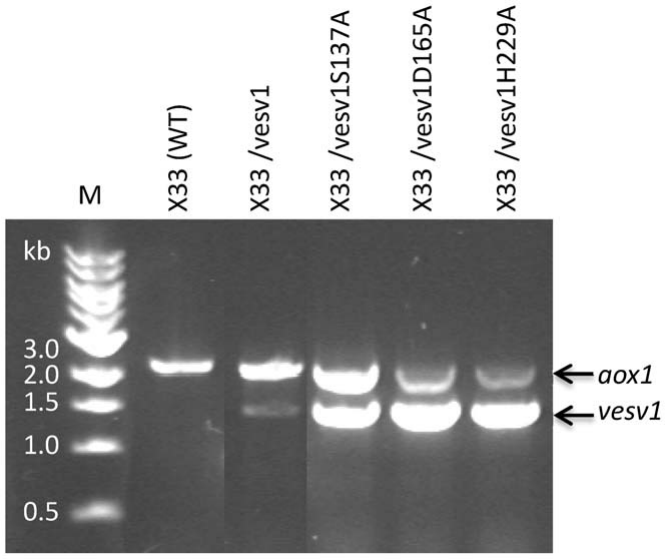
Expression analysis of the active and mutated *Vesv1* forms by RT-PCR. RT-PCR with primers binding to AOX1 promoter and AOX1 terminator was performed on total RNA of wild type *P. pastoris* and of *P. pastoris* expressing active and mutated versions of *Vesv1* gene, all the strains were induced on methanol for 24 hours. The band of 2.2 kb corresponds to the native alcohol oxidase AOX1 gene, which is expressed during growth on methanol. The 1.4 kb-band corresponds to the recombinant *Vesv1* transcript. In parallel to RT-PCR, identical control reactions were carried out without addition of reverse transcriptase. There were no bands detected in control reactions (not shown) confirming the absence of DNA contamination.

To ensure that *Vesv1* gene was transcribed, we performed RT-PCR (Figure 4) and could show that the gene was expressed, though at a lower level than the mutated forms of *Vesv1* if AOX1 expression is taken as a reference for comparison. The strains expressing the active form of phospholipase A1 grew poorly on methanol-containing plates in spite of the presence of the active AOX1 gene as confirmed by colony PCR. We attempted high-cell density cultivation of a *P. pastoris* strain expressing active *Vesv1* gene to see if higher yields could be obtained, however the culture went into growth arrest at the switch to the inducing substrate methanol even though the substrate was introduced at very low feed rate (data not shown).

The mutated versions of rVes v 1 of the expected size of 36 kDa could be detected in fermentation broth by Western blot. The expression was slightly better at 20°C than at 30°C both in Mut^+^ and Mut^S^ strains as measured by ELISA. The comparison between the Mut^+^ or Mut^S^ strains is not very relevant in this contest as the optimal induction times for these two strains can differ.

It can also be concluded that the proteins with modifications S137A and D165A were in general expressed poorer than the protein with modification H229A and this also correlates with the presence of some residual phospholipase 1 activity in the fermentation broth of those two variants, while H229A protein does not have a detectable PLA1-activity. Silver-stained SDS-PAGE of the fermentation broth of KM71H strains expressing active rVes v 1 or partially active rVes v 1 D165A shows abundant protein bands, while much fewer proteins and at lower concentrations are seen for the strain expressing empty plasmid or enzymatically inactive form of Ves v 1 (data not shown). This could be a sign of cell lysis due to destruction of cell membrane by the secreted phospholipase A1.

### Purification and characterisation of enzymatically inactive rVes v 1 H229A

**Figure 5:**
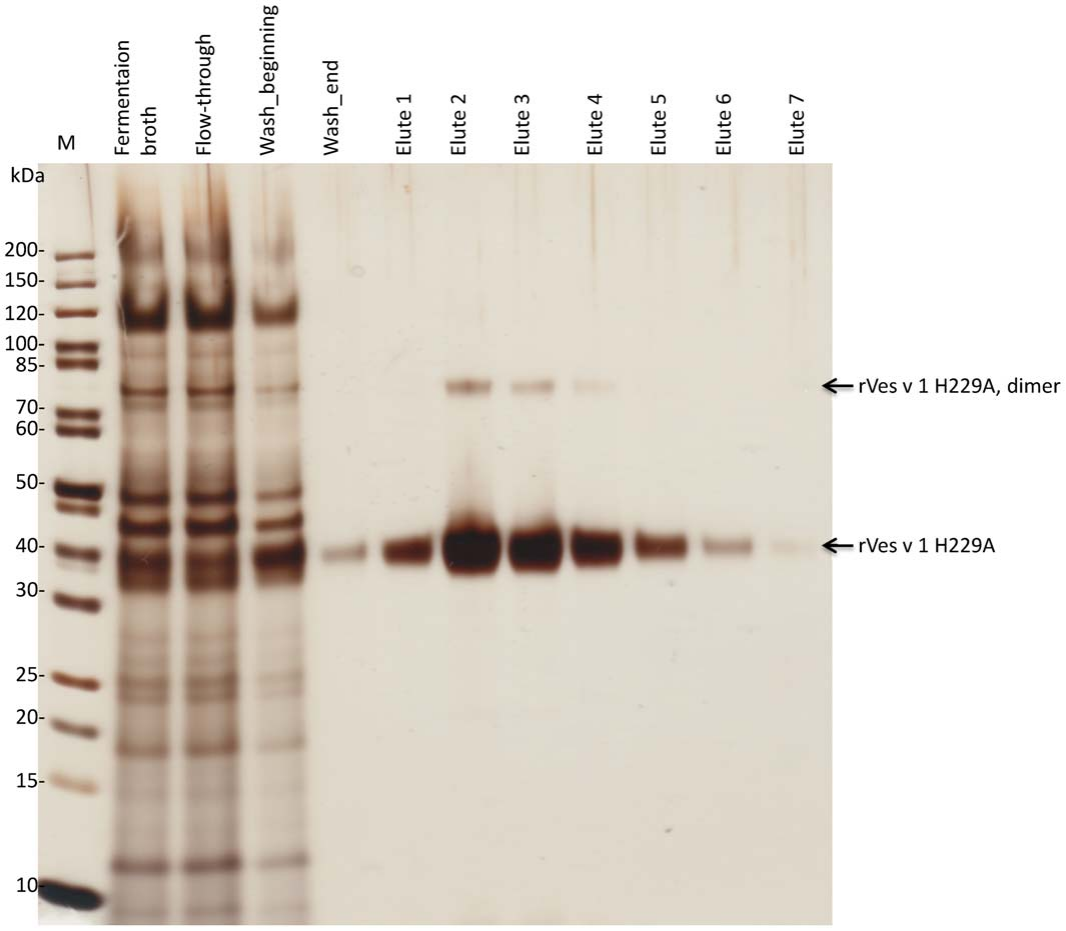
Purification of rVes v 1 H229A. The enzymatically inactive protein was purified from *P. pastoris* fermentation broth using Ni-affinity chromatography. 10 µl samples of the fermentation broth, flow-through, wash and elution fractions were separated on SDS-PAGE and silver-stained. The size marker is 5 µl of 10-fold diluted unstained PageRuler (Fermentas).

**Figure 6:**
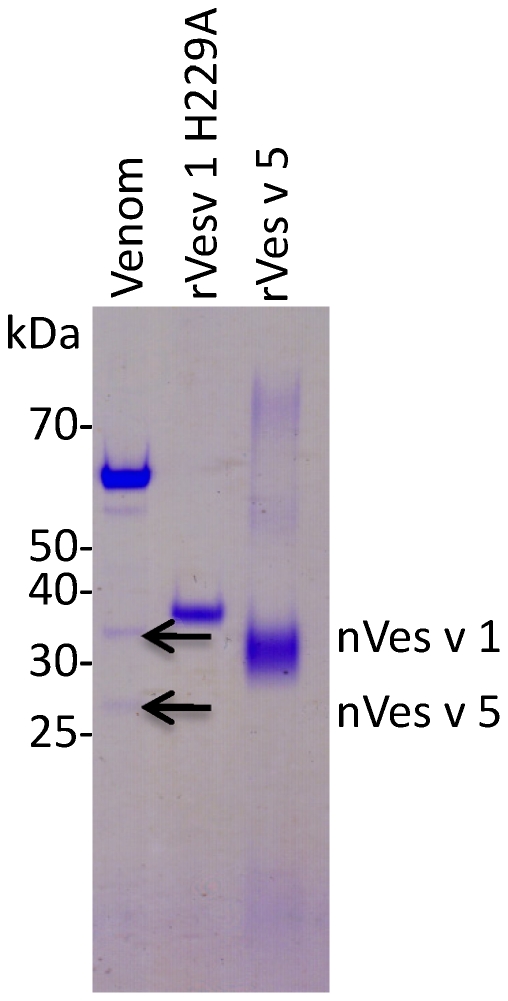
SDS-PAGE gel of venom and purified rVes v 1 H229A and rVes v 5. 1,000 SQ units of venom extract and 300 ng of purified rVes v 1 H229A and rVes v 5 were separated on SDS-PAGE and stained with coomassie. The identity of recombinant allergens and of the native nVes v 1 and nVes v 5 proteins in the venom was confirmed by MALDI-TOF MS.

We purified the rVes v 1 protein with mutation H229A from fermentation broth of 120-hour shake flask culture of KM71H strain. 1.7 mg/L was purified using Ni-affinity chromatography (Figure 5). The purification conditions were quite stringent as we wanted to obtain as pure protein as possible, otherwise higher yield could be obtained. Besides the 36 kDa protein band corresponding to rVes v 1 H229A monomer, a weak 72 kDa band was observed in a few elution fractions with the highest protein concentration. This is most likely a protein dimer appearing due to inter-disulphide bridge formation. The 72-kDa artefact disappeared after protein desalting and dilution in storage buffer (Figure 6). The 36 kDa protein was analyzed by MALDI-TOF MS (Figure 6) and was identified as *V. vulgaris* phospholipase A1 with 10 independent peptides matching with a mass tolerance of 50 ppm and with protein sequence coverage of 39% (p<0.05). Several bands in the wasp venom extract were analyzed by MALDI-TOF MS as well and the bands corresponding to the native phospholipase A1 and antigen 5 were found with significance p<0.05 (Figure 6). The recombinant proteins have a size 2.7 kDa larger than the native allergens due to the presence of the C-terminal tag.

### IgE binding of rVes v 1 H229A

**Figure 7:**
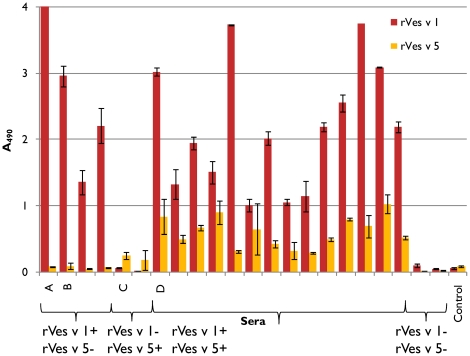
Binding of IgE antibodies from allergic patients sera to rVes v 1 H229A and rVes v 5 as measured by EAST. EAST results are shown for twenty two sera. 5 sera (A, B, C, D and control serum) were chosen for further studies. Two sera (A and B) showed a positive response to rVes v 1 H229A and negative to rVes v 5, one serum (C) showed the contrary, serum D was positive for both allergens, while control serum did not react with either of the allergens.

The ability of rVes v 1 H229A to bind specific IgE antibodies was investigated in EAST assays with sera from the wasp-allergic patients. The presence of antigen 5-specific IgEs was tested as well. Twenty two sera were selected that had a high reactivity to wasp venom and no reactivity to honey-bee venom in ImmunoCAP tests. By choosing the sera that did not cross-react with honey-bee venom we avoided the interference of specific IgE antibodies directed towards carbohydrate determinants. Out of 22 sera 14 (64%) reacted with both rVes v 1 H229A and antigen 5, 4 (18%) reacted with only Ves v 1 H229A, 2 (9%) with only antigen 5 and 2 (9%) did not react with either of the allergens (Figure 7). The latter group of patients could be sensitized to other components of the wasp venom as the peptide bone of hyaluronidase or dipeptidyl peptidase IV. The control non-allergic sera did not react with either of the recombinant allergens.

**Figure 8:**
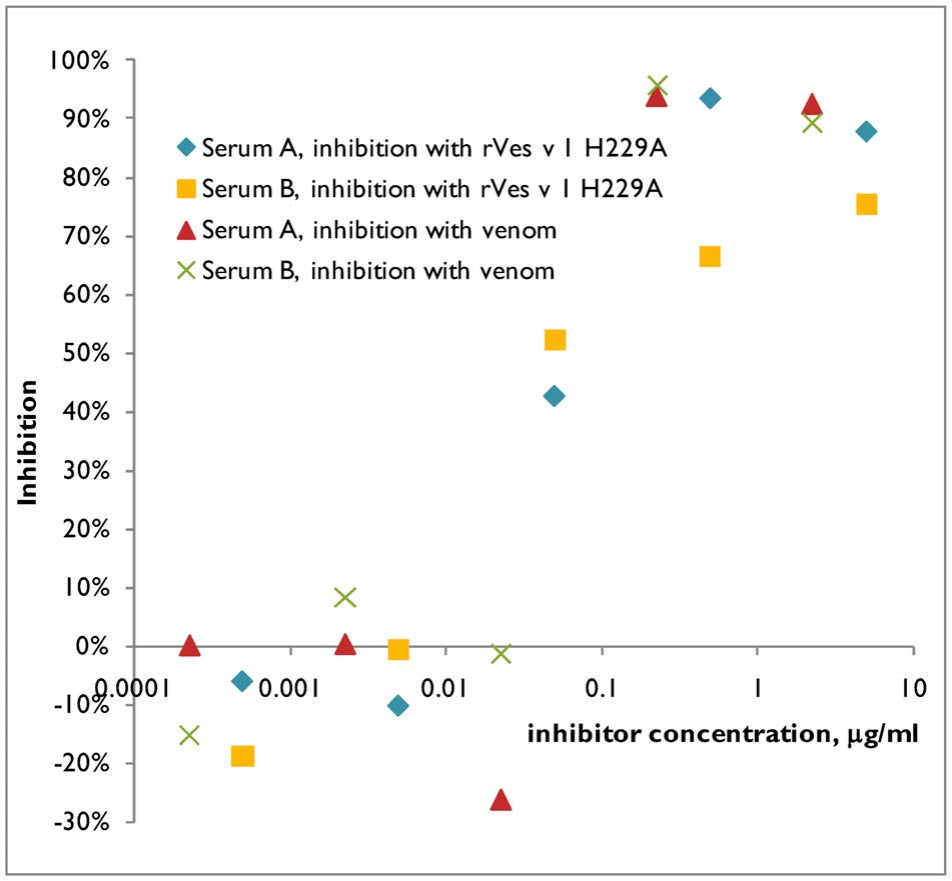
Inhibition of binding of two Ves v 1-allergic sera to wasp venom extract by rVes v 1 H229A. Microtiter plates were coated with wasp venom and were incubated with sera mixed with different concentrations of either wasp venom or rVes v 1 H229A to test their ability to inhibit the binding. Two sera A and B, which showed positive response to rVes v 1 H229A and negative to rVes v 5 in EAST assay, were chosen. The inhibition percent shows the decrease of absorbance in comparison to the sample where no inhibitor was present. The shown inhibition values are averages of two replicates. The absorbance A_490_ values for non-inhibited samples were 1.1 for serum A and 0.9 for serum B.

Two sera (A and B), which reacted with rVes v 1 H229A, but not with rVes v 5, were chosen for inhibition study, where the plates were coated with venom extract and the binding of sera to the venom was inhibited with different concentrations of recombinant rVes v 1 H229A (Figure 8). Binding of serum A to wasp venom was completely inhibited both by venom itself and by rVes v 1 H229A. For serum B the maximal inhibition by rVes v 1 H229A was 75%, whereas venom gave 96% inhibition.

### Histamine release by rVes v 1 H229A

**Figure 9:**
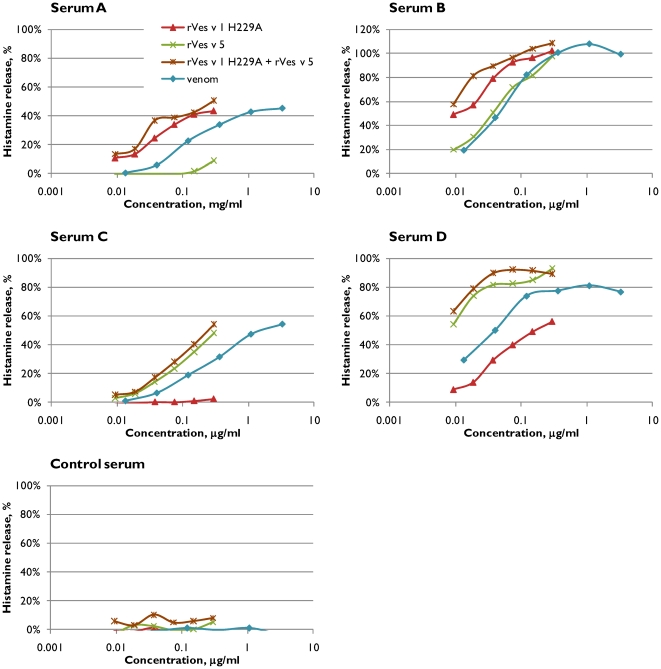
Histamine release. Histamine release from human basophils sensitized with IgE antibodies from five sera (same as on Figure 7) was tested when the basophiles were challenged with rVes v 1 H229A, rVes v 5 or wasp venom. The signal was considered positive when more than 10% of the total histamine present in the basophils was released.

Histamine release (HR) from basophils sensitised with IgE antibodies from allergic or control sera was used to test the immunological activity of the recombinant allergens rVes v 1 H229A and rVes v 5, and wasp venom (Figure 9). The histamine release assay is more sensitive than EAST inhibition and positive response (above 10% histamine release) could be detected for recombinant allergens concentrations as low as 10 ng/ml. As illustrated in Figure 9, serum A showed histamine release with rVes v 1 H229A and venom, but not with rVes v 5. Serum B, however, showed release with both allergens, indicating that the lack of detection of rVes v 5 response in EAST attributes to the lower sensitivity of the assay. Reactivity of serum B IgE antibodies towards rVes v 5 explains why the binding to venom could not be completely inhibited with rVes v 1 H229A in the EAST inhibition assay. Serum C, which was characterized as rVes v 1 H229A-negative, rVes v 5-positive in the EAST assay, behaved in the same way in HR assay mediating HR with only Ves v5 and venom. HR results for the Ves v1 H229A and Ves v 5 double positive serum (D) and for the control serum were also consistent with EAST results, showing response for both recombinant allergens and venom with serum D, but no HR with control serum, respectively.

## Discussion

Specific immunotherapy (SIT) is used for treatment of various allergies, where wasp and honey bee venom allergy, birch and grass pollen allergy, house dust mites allergy are but a few examples. Conventional allergy vaccines are partially purified allergenic extracts, which can differ in batch-to-batch composition and are difficult to standardize. The recent developments in cloning and characterization of recombinant allergens have paved the way for the new generation of recombinant allergy vaccines, which can be produced with a high-consistent quality under good manufacturing practice conditions. Moreover, they allow composing patient-tailored vaccines according to patients' individual sensitization profiles. Recombinant birch pollen allergen Bet v 1 and a cocktail of recombinant grass pollen allergens have been shown to be efficient and safe for allergy treatment in human trials [28], [29]. To the best of our knowledge, there is no clinical data on recombinant venom immunotherapy, though some animal testing has been conducted showing efficacy of a honey bee fusion vaccine, combining T-cell epitopes of three allergens [30], and of wasp venom antigen 5 in murine model [31].

Availability of recombinant wasp venom allergens produced in a safe expression host is an obstacle in developing recombinant immunotherapy. While all of the allergens have been expressed in baculovirus system and are well-suited for diagnostic purposes, for therapeutic purpose it is desirable to produce the allergens in a host well-suited for industrial production. One of the major allergens from the wasp venom, antigen 5, was successfully expressed in a eukaryotic host yeast *P. pastoris* previously, and here we expressed another major allergen, phospholipase A1.

We previously found that expression of phospholipase A1 on the surface of another yeast *S. cerevisiae* causes 70–82% growth inhibition [12]. Indeed our attempts to express an enzymatically active phospholipase A1 in yeast *P. pastoris* resulted in defective cell growth on plates and lead to cell lysis upon induction of expression in fermentors. We therefore decided to express an enzymatically inactive version of the protein instead. Such a protein, presuming that its immunological and allergenic properties are preserved, might be better suited for immunotherapy as it would not cause side-effects due phospholipids degradation. We performed single point mutations replacing the amino acids in the predicted active site of the protein with alanine. The 3D modeling of the active and mutated molecules did not show any significant changes of the protein structure due to the mutations. Three mutated versions of the allergen were expressed in *P. pastoris*. One of the variants, with mutation H229A, had no detectable phospholipase A1 activity and was secreted by P. pastoris at higher yields than other two forms. Although enzymatically inactive, the protein preserved its IgE binding activity as shown in EAST and inhibition EAST using serum from wasp venom allergic patients, it was also immunological active illustrated by its capability to mediated histamine release from basophils sensitized with wasp venom specific IgE.

The yield of pure recombinant protein was 1.7 mg/L fermentation broth. The yield can be further enhanced by strain improvement, where either a rational approach can be undertaken with optimization of promoter, signal sequence, copy number in the genome or a simple screening of a larger number of clones can be carried out. The fact that expression of Ves v 1 was higher at lower temperature (20°C instead of 30°C) indicates possible limited capacity of the cells to properly fold the given protein. If this is the case, usage of a weaker promoter could be an advantage as this would prevent the occurrence of the unfolded protein response. Furthermore fermentation can be optimized, where high cell-density cultivations can routinely give order of magnitude higher yields than shake flask cultures.

In conclusion, we established expression of an enzymatically inactive wasp venom allergen rVes v 1 in methylotrophic yeast *P. pastoris*. The protein had histidine 229 in the enzyme active site replaced by alanine. The protein showed immunological activity in EAST and histamine release assay and could inhibit the binding of Ves v 1-reactive sera to wasp venom. It presents a candidate of recombinant immunotherapy, particularly in combination with another major wasp venom allergen rVes v 5, which has also been produced in *P. pastoris*.
